# Maternal Vitamin D Status and Its Association with Neonatal Health: Clinical Implications and Influencing Factors

**DOI:** 10.3390/nu17172761

**Published:** 2025-08-26

**Authors:** Andreea Bianca Stoica, Maria Oana Săsăran, Claudiu Mărginean

**Affiliations:** 1 Department of Pediatrics 1, “George Emil Palade” University of Medicine, Pharmacy, Science and Technology of Targu Mures, Gheorghe Marinescu Street No. 38, 540136 Targu Mures, Romania; andreeabstoica9@gmail.com; 2 Department of Pediatrics 3, “George Emil Palade” University of Medicine, Pharmacy, Sciences and Technology of Targu Mures, Gheorghe Marinescu Street No. 38, 540136 Targu Mures, Romania; 3 Department of Obstetrics and Gynecology 2, “George Emil Palade” University of Medicine, Pharmacy, Sciences and Technology of Targu Mures, Gheorghe Marinescu Street No. 38, 540136 Targu Mures, Romania; marginean.claudiu@gmail.com

**Keywords:** vitamin D, pregnancy, supplementation, neonatal vitamin D, maternal-fetal health

## Abstract

**Introduction:** Adequate maternal vitamin D status during pregnancy is essential for fetal skeletal development and neonatal vitamin D reserves. Evidence from Central and Eastern Europe on maternal deficiency, its determinants, and supplementation effectiveness in late pregnancy is limited. This study assessed the impact of 2000 IU/day and 4000 IU/day maternal vitamin D supplementation during the third trimester, compared to no supplementation, on maternal and neonatal 25-hydroxyvitamin D [25(OH)_2_D] levels at birth, and explored sociodemographic, obstetric, dietary, and lifestyle factors affecting vitamin D status. **Methods:** In a cross-sectional study at Târgu Mureș County Clinical Hospital, Romania, 322 term mother–newborn pairs (37–41 weeks) from January 2021 to July 2023 were evaluated. The maternal and umbilical cord 25(OH)_2_D was measured via electrochemiluminescence immunoassay. Data on socioeconomic status, parity, sun exposure, diet, and supplementation were collected through questionnaires and records. Statistical analysis included chi-square, linear regression, and multivariate modeling. **Results:** Vitamin D insufficiency and deficiency affected 32.3% and 18.9% of mothers, respectively. Supplementation was the strongest predictor of sufficiency (*p* < 0.01), showing a dose–response effect (r = 0.84, *p* < 0.01). Maternal and neonatal 25(OH)_2_D levels were strongly correlated (r = 0.99, *p* < 0.01). Although several factors correlated with deficiency in univariate analyses, only supplementation remained significant in multivariate models. No link was found between 25(OH)_2_D status and neonatal anthropometrics or early complications. **Conclusions:** A high prevalence of vitamin D deficiency has been documented among pregnant women in Romania. High-dose supplementation during late pregnancy is critical to ensure sufficient maternal and neonatal vitamin D, highlighting the need for standardized antenatal supplementation protocols, especially in disadvantaged groups.

## 1. Introduction

Vitamin D is a fat-soluble secosteroid essential for calcium and phosphorus homeostasis, bone development, and mineralization, while also modulating immune and endocrine functions. As it can be sequestered in adipose tissue rather than circulating in free form, its bioavailability may vary. Maternal 25(OH)_2_D readily crosses the placenta and represents the primary source of vitamin D for the fetus [1]. Vitamin D is obtained from two major sources: endogenous synthesis in the skin via ultraviolet B (UVB) radiation exposure, and dietary intake [2]. While natural food sources such as fatty fish, fish liver oils, eggs, and beef liver contribute to 25(OH)_2_D status, their content is typically insufficient to meet daily requirements [3]. Consequently, many countries have introduced vitamin D fortification programs, especially in populations at higher risk of deficiency due to limited sunlight exposure [4].

The 25(OH)_2_D status is also influenced by socioeconomic and environmental factors. Studies have shown that education, employment status, and household income influence healthcare-related awareness and decision-making, and that a lower socioeconomic status may lead to vitamin D deficiency [5]. The most important environmental factor related to 25(OH)_2_D status is sun exposure. Individuals with limited sun exposure due to an indoor lifestyle, mobility issues, or the use of sunscreen or protective clothing have an increased risk of vitamin D deficiency [6]. Another factor that influences 25(OH)_2_D status in women is the number of pregnancies (parity), as women with multiple pregnancies may have lower vitamin D stores, especially if pregnancies are close together [7].

Pregnancy represents a critical window during which adequate 25(OH)_2_D levels are essential for both maternal health and optimal fetal development. Hypovitaminosis D in pregnant women has been associated with increased risk of preeclampsia, gestational diabetes, and bacterial vaginosis [8]. Furthermore, insufficient maternal 25(OH)_2_D levels may impair fetal skeletal development and contribute to adverse neonatal outcomes such as a low birth weight and a higher risk of chronic diseases in later life [9]. Given that the transfer of 25(OH)_2_D to the fetus occurs mainly during the third trimester, maternal status is the key determinant of neonatal vitamin D stores at birth [10].

Despite growing awareness, there is no universal consensus regarding the optimal dose of vitamin D supplementation during pregnancy. International guidelines offer varying recommendations, ranging from 600 IU to 4000 IU daily, often without differentiation based on geographic, dietary, or individual risk factors [11,12]. Moreover, regional data on the effectiveness of these supplementation strategies, particularly in Central and Eastern Europe, remain scarce.

Pludowski et al. reviewed vitamin D status in Central Europe and showed disparities in the available reported data of various countries, as well as the variability in 25(OH)_2_D levels with the season during which these were measured [13]. In neonates, studies from Poland show particularly low mean values of 25(OH)_2_D levels, of under 20 ng/mL, prior to the initiation of supplementation, whereas maternal 25(OH)_2_D levels ranged from deficient and insufficient in the winter to normal values in the summer [13,14]. Given the higher prevalence of vitamin D deficit in Central and Eastern Europe for the general population, particular guideline recommendations have been issued for these regions [15]. Adult vitamin D supplementation in the general population at doses of 800 to 2000 IU per day is recommended. For pregnant women, in whom 25(OH)_2_D status should be routinely screened, the recommended vitamin D supplemental doses range from 1500 to 2000 IU per day for countries such as Russia, Poland or Hungary [16,17,18]. In Bulgaria, the amount of daily vitamin D supplements in pregnancy is in accordance with the recommendations issued by the Endocrine Society, ranging between 600 and 2000 IU/day [19,20].

The aim of this study was to investigate the effect of a wide range of factors on the 25(OH)_2_D status of pregnant women and their newborns in the perinatal period. The investigated factors included maternal vitamin D supplementation with either 2000 IU or 4000 IU daily during the third trimester, sociodemographic characteristics (age, area of residence, educational attainment, household income, employment status), obstetric variables (gravidity, parity, gestational diabetes), lifestyle and dietary habits (fish intake, sun exposure patterns, number of pregnancy months during the high UV exposure season), and neonatal characteristics (sex, anthropometric measurements, APGAR scores, birth weight classification, and early neonatal complications). To the best of our knowledge, this is the first study to evaluate 25(OH)_2_D status among pregnant women and their newborns in a Romanian population, while also examining key influencing factors such as supplementation, diet, sun exposure, and socioeconomic background. By providing region-specific data, this study aims to add a valuable perspective to the broader understanding of maternal and neonatal 25(OH)_2_D status in Europe and to guide optimized vitamin D supplementation strategies for pregnant populations at risk of hypovitaminosis D.

## 2. Materials and Methods

### 2.1. Study Population

This cross-sectional observational study was conducted from January 2021 to July 2023 at the Obstetrics Clinic of the County Clinical Hospital of Târgu Mureș, Romania. Pregnant women in their third trimester (gestational weeks 37–41) who presented for delivery during this period were eligible for inclusion. Inclusion criteria entailed singleton pregnancies, term deliveries (≥37 gestational weeks), absence of chronic diseases or pregnancy-related complications potentially affecting vitamin D metabolism, and provision of written informed consent. In order to assess the effect of maternal supplementation on the 25(OH)_2_D status of the mothers and their newborns, we included women with no supplementation, supplementation with 2000 UI/day, or supplementation with 4000 UI/day in the last trimester. Exclusion criteria comprised preterm birth, multiple gestations, chronic renal or hepatic diseases, endocrine disorders (including thyroid dysfunction and diabetes mellitus), autoimmune diseases, malabsorption syndromes, hematologic disorders, ongoing therapy with agents that may alter vitamin D metabolism (e.g., corticosteroids, antiepileptic agents), and insufficient serum volume for 25(OH)_2_D measurement. Women who used vitamin D supplementation with doses other than 2000 UI/day or 4000 UI/day were also excluded. The final analysis comprised 322 mother–newborn pairs meeting all inclusion criteria.

### 2.2. Data Collection and Laboratory Methods

Maternal information was collected using standardized questionnaires and verified through medical record review. The variables assessed included maternal age, area of residence (urban or rural), educational attainment (middle school/high school/university), household income, employment status, parity (1 child/2 children/>2 children), smoking status, presence of gestational diabetes, and vitamin D supplementation status with dosage. Lifestyle and dietary factors were also documented, encompassing weekly fish consumption, sun exposure patterns (duration of exposure, type of clothing worn, and sunscreen use), and the number of pregnancy months overlapping with the high UV exposure period (from May to September).

Neonatal data were systematically collected and included gestational age at birth, sex, and detailed anthropometric measurements (birth weight, crown–heel length, and head circumference). APGAR scores were assessed at 1 and 5 min postpartum. Birth weight was subsequently classified relative to gestational age into three categories: small for gestational age (SGA), appropriate for gestational age (AGA), or large for gestational age (LGA). Additional perinatal information encompassed the mode of delivery, type of feeding at hospital discharge, administration of vitamin K prophylaxis, neonatal vaccination status, occurrence of early neonatal complications (including jaundice, infections, or hematoma), administration of antibiotic therapy, and total duration of hospitalization.

Upon admission, 10 mL of maternal venous blood was collected, and 5 mL of umbilical cord blood was drawn immediately following childbirth. Blood was drawn into serum-separating Vacutainer tubes and allowed to clot at room temperature for 15 min. Subsequently, samples were centrifuged at 3000 rpm for 2 min to separate the serum, which was then aliquoted and stored at −20 °C until analysis.

Serum levels of 25(OH)_2_D were determined using the electrochemiluminescence immunoassay (ECLIA) method on a Cobas e402 analyzer (Roche Diagnostics GmbH, Mannheim, Germany). The technique is based on a competitive binding reaction, in which 25(OH)_2_D and 25(OH)_3_D are first released from their endogenous carrier proteins through a specific pretreatment step. These free forms of vitamin D subsequently bind to a ruthenium-labeled vitamin D binding protein, forming a detectable complex. The method incorporates monoclonal antibodies to selectively limit interference from structurally similar metabolites, including 24,25-dihydroxyvitamin D. Detection is achieved via a chemiluminescent signal that is inversely proportional to the concentration of 25(OH)_2_D, calibrated against a reference curve created with biotin-labeled reagents and streptavidin-coated microparticles.

Vitamin D insufficiency was defined as serum 25(OH)_2_D levels of 20–29.9 ng/mL and deficiency as serum 25(OH)_2_D levels of <20 ng/mL. Serum 25(OH)_2_D levels of 30 ng/mL or more were considered normal. These definitions are in line with the guidelines of the Endocrine Society, available at the moment of patient enrollment [20].

#### Ethical Statements

All study procedures complied with the ethical standards established by the Declaration of Helsinki. Prior to enrollment, all participating women provided written informed consent for themselves and their newborns. The study protocol received approval from the Ethics Committee of Mureș County Clinical Hospital (approval no. 382, 9 December 2020) as well as from the Ethics Committee of the “George Emil Palade” University of Medicine, Pharmacy, Science, and Technology of Târgu Mureș (approval no. 1227, 18 December 2020).

### 2.3. Statistical Analysis

Statistical analyses were conducted using GraphPad Prism, version 9.0.2 (GraphPad Software, Boston, MA, USA). The dataset was summarized using descriptive statistical methods, including means with standard deviations (SD), medians, and proportions, as appropriate. The normality of continuous variables was assessed using the Kolmogorov–Smirnov test.

For comparisons between groups, Student’s *t*-test was applied to normally distributed variables, whereas the Mann–Whitney U test was used for variables that deviated from normality. Categorical variables were analyzed using the chi-square (χ^2^) test. Relationships between continuous variables were examined via Pearson or Spearman correlation coefficients, contingent on data distribution. Additionally, linear regression analysis was conducted when appropriate to evaluate associations.

Statistical significance was defined as a two-tailed *p*-value less than 0.05, with all outcomes reported alongside 95% confidence intervals (CI).

## 3. Results

This study included 322 pregnant women in their third trimester (37–41 weeks of gestation), stratified into three groups according to serum maternal 25(OH)_2_D status: normal levels (*n* = 157; 48.7%), insufficiency (*n* = 104; 32.3%), and deficiency (*n* = 61; 18.9%). The mean maternal age did not differ significantly among groups (29.92 ± 4.96 years vs. 29.37 ± 6.02 years vs. 28.85 ± 6.11 years, respectively; *p* = 0.46). A higher proportion of women with vitamin D sufficiency resided in urban areas compared to those with insufficiency or deficiency (*p* = 0.04). The demographic and socioeconomic characteristics, as well as the dietary and environmental exposure patterns of the mothers included in the study are presented in Table 1.

Socioeconomic factors were strongly associated with maternal 25(OH)_2_D status. Women with insufficient or deficient levels were significantly less likely to have completed higher education (73.9% of women with sufficiency vs. 29.6% of women with deficiency; *p* < 0.01) and more frequently reported low or very low household income (42.6% of women with deficiency vs. 17.8% of women with sufficiency; *p* < 0.01). Unemployment was also markedly higher among women with deficiency (42.6%) compared to those with normal levels (16.6%; *p* < 0.01).

Gravidity and parity exhibited an inverse relationship with maternal 25(OH)_2_D levels. Multiparity (≥2 children) was more prevalent among those with deficiency (42.6%) than those with sufficiency (11.5%), and the mean number of gestations increased progressively across groups (1.84 ± 1.34 among women with sufficiency vs. 2.91 ± 2.29 among women with deficiency; *p* < 0.01). This association was further supported by Spearman correlation analysis (r = −0.36; *p* < 0.01).

Lifestyle factors were also significantly linked to maternal 25(OH)_2_D status. Regular fish consumption was reported by 62.4% of women with normal 25(OH)_2_D levels compared to 27.9% of those with deficiency (*p* < 0.01). Most participants reported limited sun exposure with high body coverage, and sunscreen use was more frequent among women with low 25(OH)_2_D status (29.5% vs. 12.1% of women with sufficiency; *p* < 0.01).

Vitamin D supplementation during pregnancy emerged as the strongest determinant of maternal 25(OH)_2_D status. All women with adequate 25(OH)_2_D levels reported taking supplements, while none of the women with deficiency had received supplementation (*p* < 0.01). Moreover, a positive dose–response relationship was observed between the daily supplementation dose (2000 IU vs. 4000 IU) and maternal 25(OH)_2_D concentrations (r = 0.84; *p* < 0.01).

We found a strong positive correlation between maternal and neonatal 25(OH)_2_D concentrations (r = 0.99; *p* < 0.01). In contrast, neonatal anthropometric measures (birth weight, length, head circumference), sex distribution, APGAR scores, and early neonatal complications (jaundice, infection) did not differ significantly among groups (all *p* > 0.05). However, neonates born to vitamin D-deficient mothers had markedly lower serum 25(OH)_2_D concentrations at birth (17.32 ± 2.97 ng/mL) compared to those born to mothers with adequate 25(OH)_2_D status (48.82 ± 10.99 ng/mL; *p* < 0.01) (Table 2).

**Figure 1:**
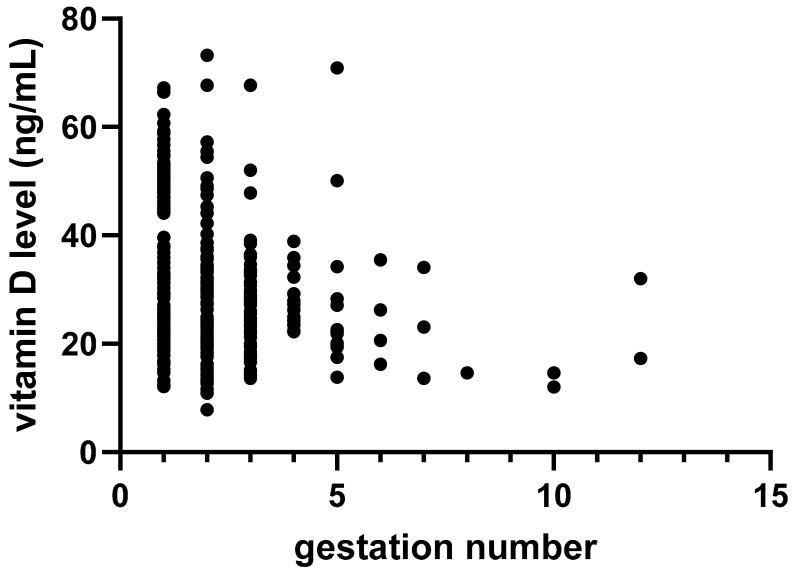
Linear regression correlation between gestation number and maternal serum vitamin D levels.

Several numerical factors that could influence maternal 25(OH)_2_D levels were analyzed using linear regression. A non-parametric Spearman analysis showed a moderate inverse correlation between 25(OH)_2_D levels and the number of pregnancies (r = −0.36; 95% CI −0.45 to −0.25; *p* < 0.01; Figure 1).

**Figure 2:**
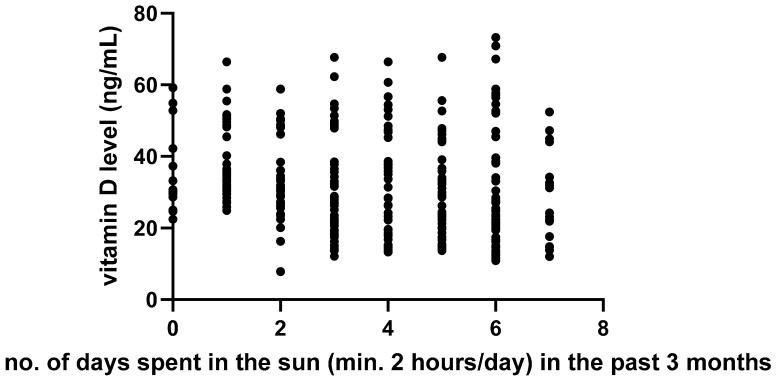
Association between sun exposure and maternal 25(OH)_2_D levels as determined by linear regression analysis.

We also examined whether sun exposure (measured as the average number of days per week with at least 2 h spent outdoors) was associated with 25(OH)_2_D levels. Interestingly, an inverse association was found (r = −0.28; 95% CI −0.38 to −0.18; *p* < 0.01; Figure 2), suggesting that self-reported sun exposure may not reliably reflect effective UVB exposure, possibly due to clothing coverage or limited skin area exposed.

**Figure 3:**
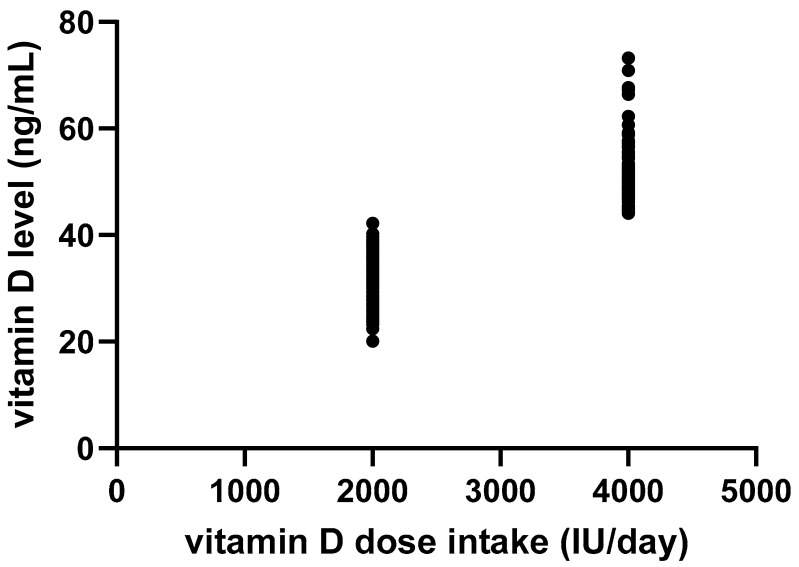
Linear regression analysis of the association between daily vitamin D intake and maternal serum vitamin D concentrations.

We also explored the potential influence of seasonality by assessing the number of sunny months during each pregnancy. The analysis revealed no statistically significant correlation with maternal 25(OH)_2_D levels (*p* = 0.54; Figure 3).

**Figure 4:**
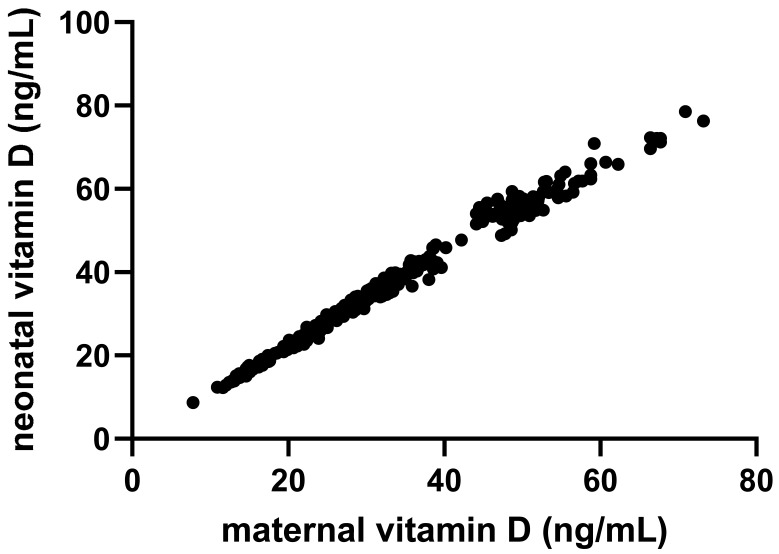
Linear regression correlation between maternal and neonatal serum vitamin D levels.

In pregnant women receiving vitamin D supplementation, higher daily doses were strongly correlated with increased serum 25(OH)_2_D concentrations (r = 0.84; 95% CI: 0.79–0.87; *p* < 0.01), demonstrating a clear dose–response relationship. Maternal and neonatal 25(OH)_2_D levels were also very closely correlated (r = 0.99; 95% CI 0.992 to 0.995; *p* < 0.01; Figure 4), reinforcing the well-established role of maternal status in determining neonatal vitamin D at birth.

To further explore the role of potential confounding factors, we performed multiple linear regression analyses. Variables such as number of pregnancies, parity, maternal age, and number of sunny months were first analyzed individually, and then combined (Table 3). While none of the variables alone showed a significant correlation with maternal 25(OH)_2_D levels, the combined model (including parity, gravidity, and seasonality) did show a significant overall association (*p* < 0.01), suggesting that their interaction may influence 25(OH)_2_D status.

A second regression analysis focused on binary variables: urban vs. rural residence, employment status, fish intake, and vitamin D supplementation (Table 4). Among these, only supplementation remained independently associated with maternal 25(OH)_2_D levels (*p* < 0.01), while the other factors showed no significant effect, either individually or in combination.

## 4. Discussion

This study provides an evaluation of 25(OH)_2_D status among pregnant women and their neonates in a Romanian clinical cohort and assesses a series of influencing factors such as supplementation habits, dietary intake, sun exposure, socioeconomic status, and parity. While many of the associations observed in this study are consistent with the existing literature, the value of our findings lies in providing the first data of this kind from Romania. Furthermore, the study distinguishes through the high number of socioeconomic and lifestyle-related characteristics, investigated as possible confounders of maternal vitamin D levels. Despite the high prevalence of vitamin D deficiency in the general population, no previous studies have systematically examined 25(OH)_2_D status in pregnant women and their newborns in this setting. These findings offer a local evidence base that can inform national public health recommendations, antenatal care strategies, and future research in Romania and similar populations in the region.

In our cohort, vitamin D insufficiency was identified in 32.3% of pregnant women, and deficiency in 18.9%. Although data on the 25(OH)_2_D status of pregnant women in Europe are scarce, a study conducted in Switzerland found a 53% prevalence of vitamin D deficiency in late pregnancy [21], and a study conducted in Belgium reported a 47–60% prevalence of vitamin D deficiency in the first trimester [22]. Regarding the prevalence of vitamin D deficiency in Romania, large studies conducted in recent years reported figures ranging from around 24.8% [23] to 55.6% [24]; however, these studies did not include pregnant women.

In Romania, lower vitamin D concentrations are more frequently observed in obese women, in samples collected during autumn months, and, for those living in rural areas, in cases of insufficiency. Conversely, higher educational attainment and better socioeconomic conditions are associated with a reduced likelihood of inadequate vitamin D levels, highlighting the value of tailored educational and preventive strategies. Nationally, the assessment of vitamin D status became part of the public health policy in 2019, when the Ministry of Health introduced a program to determine serum 25(OH)_2_D in specific high-risk categories, including hospitalized adults, pregnant or lactating women, newborns, and children. According to 2022 program data, nearly 40% of tested adults presented vitamin D deficiency, with a small proportion (13.4%) already receiving supplementation at the time of evaluation [23,25].

Lower vitamin D concentrations are more frequently observed in obese women, in samples collected during autumn months, and, for those living in rural areas, in cases of insufficiency. Conversely, higher educational attainment and better socioeconomic conditions are associated with a reduced likelihood of inadequate vitamin D levels, highlighting the value of tailored educational and preventive strategies. 

As expected, we found a strong positive association between maternal and neonatal 25(OH)_2_D levels. We obtained a high positive correlation coefficient between maternal and neonatal 25(OH)_2_D levels (r = 0.99), which might be explained by the measurement of maternal 25(OH)_2_D levels at a short time before birth and the depiction of neonatal 25(OH)_2_D status from the umbilical cord. Moreover, neonates born to vitamin D-deficient mothers had significantly lower 25(OH)_2_D levels than those born to insufficient or sufficient mothers. This finding is consistent with the relationship between maternal and neonatal 25(OH)_2_D levels described in the literature [12] and confirms that maternal status is the single most important determinant of fetal vitamin D stores at birth, due to the placental transfer of 25(OH)_2_D during gestation.

Among all the investigated factors, vitamin D supplementation was most clearly associated with maternal vitamin D sufficiency. Women in the sufficient group reported more frequent supplement intake and higher daily doses, and higher serum levels were observed in women reporting higher doses. These results are in line with previous studies indicating that supplementation during pregnancy is associated with higher maternal and neonatal 25(OH)_2_D levels [26].

Regular fish intake was also more frequent in women with sufficient 25(OH)_2_D status. While oily fish is a valuable dietary source, vitamin D content from food is usually not enough to meet the recommended intake during pregnancy [20]. This suggests that supplementation plays a central role, particularly in Romania, where studies report that the median daily vitamin D intake is well below the 400 IU/day recommendation [27].

Our multivariate models also confirmed that vitamin D supplementation was the only independent factor significantly associated with maternal 25(OH)_2_D levels. The significant impact of maternal supplementation on neonatal 25(OH)_2_D status has been previously demonstrated within a randomized controlled trial performed in Denmark, which showed that supplements of 3600 IU/day during pregnancy can lead to positive outcomes [28]. Other variables, including urban residence, employment, fish intake, parity, and seasonality, did not show consistent effects once supplementation was accounted for. This reinforces findings from other studies [26,29] and suggests that regular supplementation is linked to better 25(OH)_2_D status and may be a factor in reducing disparities related to education, income, or access to vitamin D-rich foods.

Regarding neonatal outcomes, we found no significant association between 25(OH)_2_D levels and anthropometric measures such as birth length, head circumference, or birth weight. Similarly, other parameters, including weight-for-gestational-age classification, APGAR scores at 1 and 5 min, and the occurrence of neonatal jaundice and infection, did not differ significantly across the three investigated subgroups. These results are consistent with findings from high-quality randomized controlled trials, which have shown that vitamin D supplementation during pregnancy does not influence neonatal anthropometric parameters [30,31,32,33]. However, several systematic reviews and meta-analyses have reported associations between low neonatal 25(OH)_2_D levels and a low birth weight or smaller head circumference [34], as well as links between low maternal 25(OH)_2_D status and increased risk for low birth weight [35,36,37,38]. Taken together, these findings suggest that the relationship between 25(OH)_2_D status and neonatal anthropometric parameters remains uncertain. While current evidence suggests that vitamin D has a limited effect on birth size in well-nourished populations, further research is needed to clarify potential effects on fetal growth and early neonatal outcomes.

Significant relationships were observed between various socioeconomic determinants and maternal 25(OH)_2_D status, including both insufficiency and deficiency. These included lower educational attainment, unemployment, and lower family income. Such associations are consistent with earlier research showing that socioeconomic disadvantage is linked to reduced awareness of nutritional needs, limited access to vitamin D-rich foods or high-quality supplements, and poorer health outcomes [5,39]. We also found that vitamin D deficiency was slightly more common among women from urban areas. While urban settings typically offer better access to healthcare, they may also be associated with more sedentary lifestyles and less sun exposure, through the pollution-related filtration of UV light, which could contribute to lower 25(OH)_2_D levels [40]. Overall, these results suggest that both material resources and health-related behaviors contribute to 25(OH)_2_D status, and that targeted antenatal interventions could play an important role in reducing maternal and neonatal vitamin D deficiency.

Despite Romania’s geographical latitude and seasonal fluctuations in UVB availability, no significant association was observed between the number of sunny months during pregnancy and maternal 25(OH)_2_D concentrations. This finding may have been heavily influenced by individual behaviors, such as duration of outdoor exposure, sunscreen utilization, and clothing coverage. We observed that women with sufficient 25(OH)_2_D levels were more likely to avoid sun protection, though most still reported high clothing coverage. These behaviors may partly explain the limited effect of seasonality. Similar patterns have been reported in Middle Eastern countries, where ample sunlight does not necessarily lead to sufficient 25(OH)_2_D levels due to clothing habits and avoidance of sun exposure [41]. These findings suggest that year-round supplementation may be beneficial, regardless of season or perceived sun exposure.

Although we found no association between parity and gravidity number, which are dependent one upon the other, and maternal 25(OH)_2_D levels, we observed mean higher gravidity and parity levels in the vitamin D deficient group. This suggests a cumulative depletion of nutrient stores with each subsequent pregnancy, especially if supplementation is inconsistent or dietary intake is suboptimal. This relationship is consistent with findings by Karras et al., who reported significantly lower 25(OH)_2_D levels among women with higher parity in Mediterranean countries [42]. In addition, previous data have shown that low 25(OH)_2_D levels might account for an increase in pregnancy loss rates at early gestational ages [43]. These findings suggest that nutritional depletion over time is a relevant concern and highlight the need to consider supplementation guidance in prenatal care, especially for women with higher parity.

In recent years, the role of the vitamin D receptor (VDR) polymorphisms has been taken into account, as a possible influencing factor of 25(OH)_2_D levels. A study performed in Croatia showed an association between VDR Cdx-2 single nucleotide polymorphisms (SNP) and maternal serum vitamin D levels, as well as an association between VDR Cdx2 gene polymorphism and premature birth. However, none of the studied SNPs seemed to influence neonatal 25(OH)_2_D levels, in both preterm and term neonates [44]. Another Chinese study suggested a possible correlation between maternal and fetal VDR SNPs and the risk of gestational diabetes mellitus [45]. Furthermore, recent data suggests an interplay between neonatal SNPs, such as VDR rs2228570 and GC rs4588 and neonatal anthropometric parameters, such as birth weight and head circumference [46]. Thus, these data suggest the need for further studies analyzing the co-variance between neonatal outcomes, maternal and neonatal 25(OH)_2_D status, and the SNPs of VDR polymorphisms.

### Study Limitations

This study has several limitations that must be acknowledged. Firstly, sun exposure, dietary intake, and supplement adherence were self-reported, potentially introducing recall or social desirability biases. Secondly, our sample may not fully represent rural or underserved regions of Romania, limiting generalizability. In addition, the lack of follow-up beyond birth prevents assessment of long-term health consequences of neonatal deficiency. Despite these limitations, the findings provide a valuable contribution to the evidence base on maternal and fetal vitamin D status in Eastern Europe.

## 5. Conclusions

This study confirms that vitamin D insufficiency and deficiency are common among pregnant women in Romania and are associated with lower 25(OH)_2_D levels in newborns. Maternal supplementation was the strongest factor linked to adequate 25(OH)_2_D status and remained the only independent predictor in multivariate analysis. While regular fish intake and certain socioeconomic indicators showed associations in univariate analysis, their effects were no longer significant when supplementation was taken into account.

Our findings support the existing evidence that maternal 25(OH)_2_D levels directly influence neonatal status and highlight the role of supplementation in preventing deficiency. Although no significant associations were found between maternal or neonatal 25(OH)_2_D status and early neonatal anthropometric or clinical outcomes, the long-term health implications remain uncertain and warrant further research.

Given the high prevalence of deficiency and the limited contribution of sunlight and diet alone, routine vitamin D supplementation during pregnancy appears to be a practical and effective public health measure. Antenatal care programs should emphasize the importance of adequate vitamin D intake, with particular attention to multiparous women and those from socioeconomically disadvantaged backgrounds.

## Figures and Tables

**Table 1:** Socioeconomic, dietary, and environmental exposure characteristics among mothers with normal, sufficient, and deficient vitamin D status.

Parameter		Mothers with Normal Vitamin D Levels (*n* = 157)	Mothers with Vitamin D Insufficiency (*n* = 104)	Mothers with Vitamin D Deficiency (*n* = 61)	*p* Value
**Socioeconomic characteristics**					
Maternal age (years, mean ± SD)		29.92 ± 4.96	29.37 ± 6.02	28.85 ± 6.11	0.46
Maternal background	Urban (*n*)	86	41	31	0.04
	Rural (*n*)	71	63	30	
Educational level	Middle school (*n*)	3	5	8	<0.01
	High school (*n*)	38	50	35	
	University (*n*)	116	49	18	
Familial income	Very low (*n*)	9	15	11	<0.01
	Low (*n*)	19	31	15	
	Satisfactory (*n*)	129	58	35	
Work status	Employed (*n*)	141	45	35	<0.01
	Unemployed (*n*)	26	49	26	
**Lifestyle and pregnancy-related characteristics**					
Parity	1 child (*n*)	100	35	15	<0.01
	2 children (*n*)	39	45	32	
	>2 children (*n*)	18	14	14	
Gravidity (*n*, mean ± SD)		1.84 ± 1.34	2.47 ± 1.32	2.91 ± 2.29	<0.01
Weekly fish consumption	Yes (*n*)	98	52	17	<0.01
	No (*n*)	59	42	44	
Type of sun exposure	Sunbathing (*n*)	1	5	5	<0.01
	Summer clothing (*n*)	29	24	28	
	Clothes with full coverage (*n*)	127	75	28	
Use of sunscreen	Yes (*n*)	19	13	18	<0.01
	No (*n*)	138	91	43	
Gestational age (weeks, mean ± SD)		39.08 ± 1.02	38.93 ± 1.09	38.77 ± 0.92	0.18
Smoker status	Yes (*n*)	25	24	9	0.25
	No (*n*)	132	80	52	
Vitamin D supplementation during pregnancy	Yes, 2000 IU/day (*n*)	76	56	0	<0.01
	Yes, 4000 IU/day (*n*)	81	0	0	
	No (*n*)	0	48	61	
**Delivery and laboratory findings**					
Type of delivery	Vaginal (*n*)	81	56	33	0.91
	Cesarean section (*n*)	76	48	28	
Serum 25(OH)_2_D concentrations (ng/mL, mean ± SD)		48.82 ± 10.99	27.92 ± 3.59	17.32 ± 2.97	

**Table 2:** Neonatal anthropometric measures and complications across maternal groups with normal, sufficient, and deficient vitamin D levels.

Neonatal Parameter		Mothers with Normal Vitamin D Levels (*n* = 157)	Mothers with Vitamin D Insufficiency (*n* = 104)	Mothers with Vitamin D Deficiency (*n* = 61)	*p* Value
Sex	Female (*n*)	74	46	28	0.89
	Male (*n*)	83	58	33	
Length (cm)		53.20 ± 2.37	53.36 ± 2.39	53.26 ± 2.24	0.91
Head circumference (cm, mean ±SD)		33.86 ±1.58	33.92 ± 1.34	33.90 ± 1.41	0.95
Birth weight (g, mean ± SD)		3319 ± 487.5	3345 ± 489.7	3353 ± 465	0.91
Weight for gestational age	SGA (*n*)	14	9	8	0.8
	AGA (*n*)	129	83	48	
	LGA (*n*)	14	12	5	
APGAR score at 1 min (mean ± SD)		9.29 ± 0.91	9.35 ± 0.83	9.47 ± 0.78	0.29
APGAR score at 5 min (mean ± SD)		9.74 ± 0.58	9.73 ± 0.50	9.82 ± 0.42	0.50
Neonatal jaundice	Yes (*n*)	77	54	22	0.21
	No (*n*)	80	60	39	
Neonatal infection	Yes (*n*)	17	12	7	0.98
	No (*n*)	140	92	54	
Vitamin D levels (ng/mL, mean ± SD)		48.82 ± 10.99	27.92 ± 3.59	17.32 ± 2.97	<0.01

**Table 3:** Assessment of individual and combined continuous predictors of maternal serum vitamin D concentrations using multiple linear regression.

Parameter	Variable	Estimate	Standard Error	95% CI	t Value	*p* Value
β0	Intercept	25.46	29.53	−32.64 to 83.57	0.8623	0.3892
β1	Gravidity	23.55	19.26	−14.34 to 61.45	1.22	0.22
β2	Parity	−24.09	27.84	−78.88 to 30.70	0.86	0.38
β3	Maternal age	0.45	1.02	−1.56 to 2.475	0.44	0.65
β4	No. of sunny months during pregnancy	−2.52	7.87	−18.01 to 12.97	0.32	0.74
β5	Gravidity + parity	−0.28	1.65	−3.54 to 2.96	0.17	0.86
β6	Gravidity + maternal age	−0.70	0.58	−1.85 to 0.44	1.21	0.22
β7	Gravidity + no. of sunny months during pregnancy	−7.31	4.98	−17.12 to 2.48	1.46	0.14
β8	Parity + maternal age	0.57	0.87	−1.14 to 2.30	0.65	0.51
β9	Parity + no. of sunny months during pregnancy	5.82	7.61	−9.16 to 20.81	0.76	0.44
β10	Maternal age + no. of sunny months during pregnancy	0.23	0.27	−0.31 to 0.78	0.84	0.40
β11	Gravidity + parity + maternal age	−0.004	0.04	−0.08 to 0.07	0.11	0.90
β12	Gravidity + parity + no. of sunny months during pregnancy	0.65	0.24	0.16 to 1.14	2.63	<0.01
β13	Gravidity + maternal age + no. of sunny months during pregnancy	0.17	0.15	−0.13 to 0.47	1.12	0.26
β14	Parity + maternal age + no. of sunny months during pregnancy	−0.23	0.23	−0.70 to 0.23	0.98	0.32

**Table 4:** Evaluation of individual and combined categorical predictors of maternal serum vitamin D concentrations using multiple linear regression.

Parameter	Variable	Estimate	Standard Error	95% CI	t Value	*p* Value
β0	Intercept	18.69	2.63	13.51 to 23.88	7.09	<0.01
β1	Urban/rural background (ref. = rural)	−0.37	3.52	−7.32 to 6.56	0.10	0.91
β2	Employment status (ref. = unemployed)	0.36	4.337	−8.16 to 8.89	0.08	0.93
β3	Fish intake (ref. = no regular intake/week)	−1.09	3.86	−8.69 to 6.50	0.28	0.77
β4	Vitamin D supplement intake during pregnancy (ref. = no supplementation)	11.88	3.24	5.49 to 18.26	3.66	<0.01
β5	Urban/rural background + employment status	4.08	5.25	−6.25 to 14.42	0.77	0.43
β6	Urban/rural background + fish intake	1.78	4.792	−7.63 to 11.22	0.37	0.70
β7	Urban/rural background + vitamin D supplement intake during pregnancy	−0.88	4.37	−9.49 to 7.73	0.20	0.84
β8	Employment status + fish intake	1.41	4.98	−8.39 to 11.22	0.28	0.77
β9	Employment status + vitamin D supplement intake during pregnancy	3.43	4.88	−6.17 to 13.04	0.70	0.48
β10	Fish intake + vitamin D supplement intake during pregnancy	2.12	4.86	−7.45 to 11.71	0.43	0.66
β11	Urban/rural background + employment status + fish intake	−6.75	5.19	−16.97 to 3.46	1.30	0.19
β12	Urban/rural background + employment status + vitamin D supplement intake during pregnancy	1.45	5.25	−8.87 to 11.78	0.27	0.78
β13	Urban/rural background + fish intake + vitamin D supplement intake during pregnancy	4.35	5.31	−6.09 to 14.81	0.81	0.41
β14	Employment status + fish intake + vitamin D supplement intake during pregnancy	3.79	5.33	−6.70 to 14.29	0.71	0.47

## Data Availability

The original contributions presented in this study are included in the article. Further inquiries can be directed to the corresponding author.
